# A Comprehensive Evolutionary Analysis of the Dihydroflavonol 4-Reductase (DFR) Gene Family in Plants: Insights from 237 Species

**DOI:** 10.3390/genes16040396

**Published:** 2025-03-29

**Authors:** Senlin Luo, Shiping Wang, Ling Yang, Kaiyong Luo, Jia Cheng, Ya Ning, Yang Dong, Weibin Wang

**Affiliations:** 1 College of Food Science and Technology, Yunnan Agricultural University, Kunming 650201, China; lsl@dongyang-lab.org (S.L.); lky@dongyang-lab.org (K.L.); chenj@dongyang-lab.org (J.C.); 2 Yunnan Provincial Key Laboratory of Biological Big Data, Yunnan Agricultural University, Kunming 650201, China; jxaa174751@163.com; 3 Institute of Agro-Products of Processing and Design, Hainan Academy of Agricultural Sciences, Haikou 571100, China; yangl@dongyang-lab.org; 4 College of Science, Yunnan Agricultural University, Kunming 650201, China; ningya0428@126.com

**Keywords:** dihydroflavonol 4-reductase (DFR), Arg-DFR, gene family, evolution, flavonoids, anthocyanin

## Abstract

Background: Dihydroflavonol 4-reductase (DFR) is a key enzyme in the flavonoid biosynthetic pathway that regulates anthocyanin and proanthocyanidin accumulation in plants. Although *DFR* genes have been studied in various species, their origin of the DFR gene family, its distribution across the plant kingdom, and the reasons behind the emergence of different DFR subtypes Methods: This study performed a whole-genome analysis of *DFR* genes in 237 plant species, including algae, mosses, ferns, gymnosperms, and angiosperms, integrating phylogeny, conserved motifs, duplication mechanisms, positive selection, and expression pattern analyses. Results: These results indicate that the *DFR* gene family originated from the common ancestor of extant ferns and seed plants, and the emergence of asparagine (Asn)-type and aspartic (Asp)-type DFRs is associated with gymnosperms. Notably, we report for the first time the presence of Asn-type, Asp-type, and arginine (Arg)-type DFRs in some species, which breaks the previous notion that Arg-type DFRs are exclusive to ferns. Tandem duplication is considered the primary driving force behind the expansion of the *DFR* family and is associated with the formation of different DFR subtypes. Furthermore, Asn-type DFRs were highly expressed during the early stages of seed development, suggesting their important role in seed development. Conclusions: Overall, this study revealed the dynamic evolutionary trajectory of the *DFR* gene family in plants, providing a theoretical foundation for future research on *DFR* genes.

## 1. Introduction

Flavonoids are widely distributed among extant plant lineages and represent a significant class of secondary metabolites within the plant kingdom [1]. This group encompasses structurally distinct subclasses, including flavones, flavonols, isoflavones, anthocyanins, and proanthocyanidins [2]. Their evolutionary diversification and radiation during plant terrestrialization are strongly associated with key adaptive innovations, enabling plants to colonize and thrive in diverse land environments [3]. Anthocyanins have emerged as multifunctional compounds serving dual roles as protective phytochemicals and ecological mediators [4,5]. They contribute to the scavenging of free radicals [6], serve as a protective agent against ultraviolet light [7], enhance tolerance to low temperatures [8], and mitigate metal toxicity [9]. Furthermore, throughout plant development, anthocyanins impart vibrant colors to flowers and fruits, thereby attracting pollinators and facilitating seed dispersal [10]. This dual functionality underscores their critical importance in stress adaptation and reproductive success.

**Figure 1:**
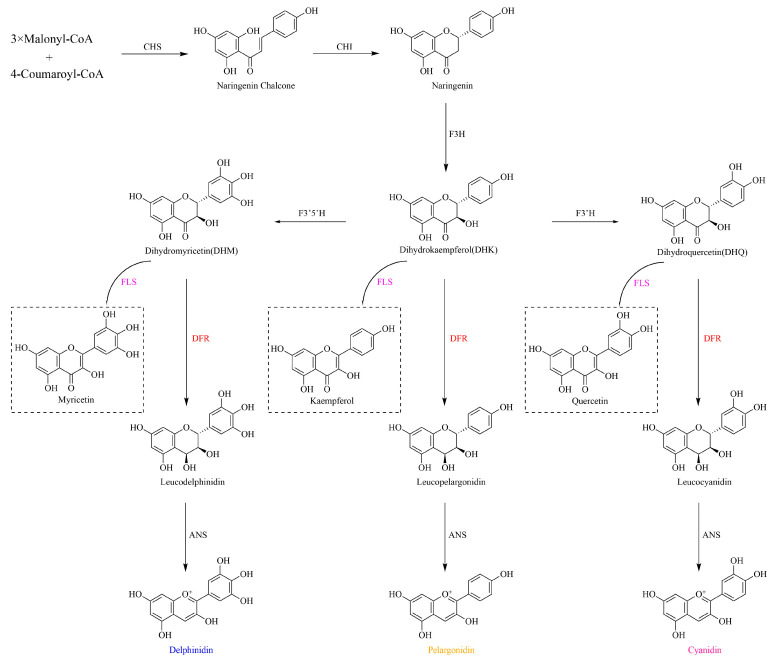
Anthocyanin biosynthesis pathways. CHS, chalcone synthase; CHI, chalcone isomerase; F3H, flavanone 3-hydroxylase; FLS, flavonol synthase; F3′H, flavonoid 3′-hydroxylase; F3′5′H, flavonoid 3′,5′-hydroxylase; DFR, dihydroflavonol 4-reductase; ANS, anthocyanin synthase. Plotted using ChemDraw (v23.2.1.1).

The first central molecule in anthocyanin biosynthesis is the branchpoint enzyme dihydroflavonol 4-reductase (DFR, EC1.1.1.219), which catalyzes the NADPH-dependent reduction in dihydroflavonols (DHFs) to leucoanthocyanidins [11,12]. Substrate specificity at this metabolic junction is governed by the competitive interplay between DFR and flavonol synthase (FLS) [13], with their relative enzymatic activities directly influencing the carbon flux partitioning between anthocyanin and flavonol production [13,14,15,16,17] (Figure 1). The existence of a substrate-specific-determining region of 26 amino acids in each DFR protein was found, from amino acids 131 to 156 [18,19]. Based on the amino acid residue at position 134 within this region, DFRs have been classified into three major types: Asn (featuring asparagine at position 134), Asp (aspartic acid), and non-Asn/Asp (neither Asn nor Asp). They have different substrate preferences, for example, the Asn-type DFR in *Gerbera hybrida* (*G. hybrida*) exhibits broad substrates for dihydrokaempferol (DHK), dihydroquercetin (DHQ), and dihydromyricetin (DHM) [20], whereas the Asp-type DFR in *Petunia hybrida* (*P. hybrida*) accepts utilizing DHQ and DHM [21]. However, the substrate preference of non-Asn/Asp-type DFRs remains undetermined. Recently, a novel type of DFR has been identified in the fern *Dryopteris erythrosora* (*D. erythrosora*), characterized by the presence of an arginine (Arg) residue at the substrate-specificity-determining site [22]. This Arg-type DFR, which is incapable of catalyzing DHM [22], represents a new classification of DFR proteins. Intriguingly, a novel Arg-type DFR identified in ferns (*D. erythrosora*) expands this classification system, exhibiting unique catalytic constraints by excluding DHM as a substrate [22].

While DFRs have been characterized in numerous plants, including *Arabidopsis thaliana* (*A. thaliana*) [23], *P. hybrida* [21], *Oryza sativa* (*O. sativa*) [24], and *G. hybrida* [20], recent studies have revealed that plant DFRs belong to a gene family. The *DFR* gene family has been identified and analyzed in various plant species, including tea [25], *Brassica napus* (*B. napus*) [26], solanaceae [27], apple [28], and strawberry [29], critical knowledge gaps persist regarding their evolutionary origins and diversification patterns across the plant kingdom. Current studies remain largely taxonomically restricted, focusing on gene family identification, phylogenetic relationships, and promoter analyses within a single species or narrow clades of closely related species. Notably, the evolutionary trajectory of DFR substrate specificity types, including the emergence of fern-specific Arg-type DFRs and ancestral origins of Asn-/Asp-type variants, remains unresolved. Consequently, this study systematically investigated the evolutionary origins, diversification patterns, and expansion mechanisms of the *DFR* gene family at the large-scale level. Our study not only elucidated the phylogenetic distribution of different types of DFR but also provides a foundation for resolving the molecular evolutionary framework of the flavonoid biosynthetic pathway.

## 2. Materials and Methods

### 2.1. Data Collection

Genome data (including General Feature Format (gff) and FASTA format (fa)) of 237 plants were collected from several public databases, including published Plant Genomes (https://www.plabipd.de/plant_genomes_pa.ep, accessed on 8 April 2024), the China National Center for Bioinformation (CNCB, https://ngdc.cncb.ac.cn, accessed on 8 April 2024), the National Center for Biotechnology Information (NCBI, https://www.ncbi.nlm.nih.gov, accessed on 8 April 2024), Phytozome (https://phytozome-next.jgi.doe.gov, accessed on 8 April 2024), Comparative Genomics (CoGe, https://genomevolution.org/coge/, accessed on 8 April 2024), Ensembl Plants (https://plants.ensembl.org/index.html, accessed on 8 April 2024), and the China National Genebank Database (CNGBdb, https://db.cngb.org, accessed on 8 April 2024). The studied species encompassed a wide range of taxa, from algae to angiosperms.

### 2.2. Construction of Species Phylogenetic Tree

First, the sequences of 237 species were aligned using BUSCO (v5.6.1) [30] to extract single-copy gene sequences with more than 80% coverage using custom Python scripts (v3.11.7). This was followed by multiple sequence comparisons of the same single-copy genes with coverage above 80% in different species using MAFFT (v7.429) [31]. Subsequently, phylogenetic analysis was performed using IQ-TREE (v1.6.11) [32] with algae as the outgroup, and 1000 replicates were set up to construct multiple single-copy gene trees. Subsequently, species phylogenetic trees were generated by merging these gene trees based on the coalescence method using ASTRAL (v5.7.8) [33]. The final calibration was based on the fossil time.

### 2.3. Identification of DFR Gene Family Members

The DFR protein sequences of *A. thaliana* (BAA85261.1) and *O. sativa* (BAA36183.1) were downloaded from the NCBI database (https://www.ncbi.nlm.nih.gov/, accessed on 20 August 2024). Subsequently, BlastP (v2.9.0+) [34] was performed on eudicots, monocots, and other taxa using the DFR protein sequences of *A. thaliana* and rice, respectively, to remove redundant protein sequences with e < l × 10^−5^. The resulting protein sequences were then further filtered using the Pfam database (PF01370) to identify and eliminate redundant sequences with e < l × 10^−5^. Finally, non-redundant sequences were verified using SMART (http://smart.embl-heidelberg.de/, accessed on 10 September 2024) and CDD (https://www.ncbi.nlm.nih.gov/Structure/bwrpsb/bwrpsb.cgi, accessed on 10 September 2024) to ensure accuracy for the obtained results. The number of DFR sequences per species was calculated using Python scripts.

### 2.4. Construction of DFR Gene Family Phylogenetic Tree

First, the DFR protein sequences of *A. thaliana*, *G. biloba*, *Bromheadia finlaysoniana* (*B. finlaysoniana*), *P. hybrid*, *Iris* × *hollandica* (*I. hollandica*), and *Nicotiana tabacum* (*N. tabacum*) were downloaded from the NCBI database as reference (three Asn-types and three Asp-types). Then, these six reference sequences, along with DFR protein sequences from 237 plants, were aligned using MAFFT (v7.429) software, and the alignment results were visualized using Jalview software (v2.11.4.1) [35]. Based on the amino acid residue at position 134, DFR sequences were classified into four types: Asn, Asp, Arg, and non-Asn/Asp/Arg. Subsequently, the six reference sequences were removed from the MAFFT output file, and the remaining sequences were trimmed using TRIMAL (v1.4) [36]. Phylogenetic trees were then constructed using IQ-TREE (v1.6.11) with the maximum likelihood (ML) method, and the number of bootstrap replicates was set to 1000. Finally, the *DFR* gene family phylogenetic tree was visualized using ChiPlot (v33.5.0) [37], and the tree was grouped according to its topology.

The GenBank accession numbers of the reference DFR protein sequences are as follows: *A. thaliana* (Asn-type), BAA85261; *G. biloba* (Asn-type), AGR34043; *B. finlaysoniana* (Asn-type), AAB62873; *P. hybrid* (Asp-type), AGI96402; *I. hollandica* (Asp-type), BAF938960; and *N. tabacum* (Asp-type), BAF96936.

### 2.5. Conserved Motif Analysis of Gene Family Sequences

Conserved motifs in the residue sequences of each DFR protein were predicted using the MEME Suite website (v5.5.7) [38] (https://meme-suite.org/meme/tools/meme, accessed on 14 October 2024), where the maximum number of motifs was set to 10 and the rest of the parameters were set to default. Motif information of the DFR proteins was plotted with the help of the website Chiplot.

### 2.6. Analysis of the Duplication Type for DFR Family Genes

The DFR sequences of 237 species were analyzed using DupGen_finder [39]. Protein sequences from multiple species were first compared using Diamond with an e value of 10^−5^ [40]. Then, duplication types were identified using the DupGen_finder-unique.pl program in DupGen_finder. This tool identifies six duplication types—whole genome duplication (WGD), tandem (TD), proximal (PD), transposed (TRD), dispersed (DRD), and singletons (SL)—by setting different outgroups for each plant taxon, and classifies each duplicated gene into one of these duplication type patterns. Python scripts were used to count the duplication types of each species and the *DFR* family genes. Significance analyses were conducted on the duplication types of whole-genome genes and *DFR* family genes using the chi-square test [41,42]. Specifically, in a Python environment, data processing was performed using Pandas, and the chi-square statistic, along with the corresponding *p*-value, was calculated using the chi2 module from the SciPy (v1.14.1) library. If the computed *p* < 0.05, it indicates that there is a significant difference in the distribution of a specific type of duplicated gene between the whole genome and the gene family; otherwise, the difference is considered insignificant.

### 2.7. Positive Selection

Homologous gene pairs were first identified using the DupGen_finder-unique.pl program in the DupGen_finder software (3.3). Subsequently, with the aid of scripts provided by Turner et al. (https://github.com/qiao-xin/Scripts_for_GB, accessed on 2 November 2024) [39], the Ka/Ks_calculator software (2.4) was used to compute the non-synonymous substitution rate (Ka), the synonymous substitution rate (Ks), and the ratio of these rates (Ka/Ks) for the identified gene pairs.

### 2.8. Gene Expression Analysis

Expression datasets containing developmental stage expression data for various tissues of *A. thaliana* and *Camelina sativa* (*C. sativa*) were retrieved from the eFP browser (https://bar.utoronto.ca/, accessed on 24 December 2024) [43]. Clustering heatmaps were subsequently generated using TBtools (v2.119) [44].

## 3. Results

### 3.1. Identification and Classification of DFR Gene Family

To conduct a comprehensive analysis of the *DFR* gene family in plants, we employed an integrative approach combining BlastP and the Hidden Markov Model (HMM). BlastP results were filtered based on a similarity (identity) threshold of 45% [45]. Through this approach, we identified *DFR* family members from 237 species, including algaes (7), bryophytes (3), ferns (5), gymnosperms (4), basal angiosperms (3), chloranthale (1), magnoliidae (1), monocots (55), and eudicots (158) (Table S1). After the exclusion of pseudogenes, a total of 745 DFR homologous sequences were obtained (Table S2). Notably, DFR proteins were present in all plant taxa except algae and mosses.

**Figure 2:**
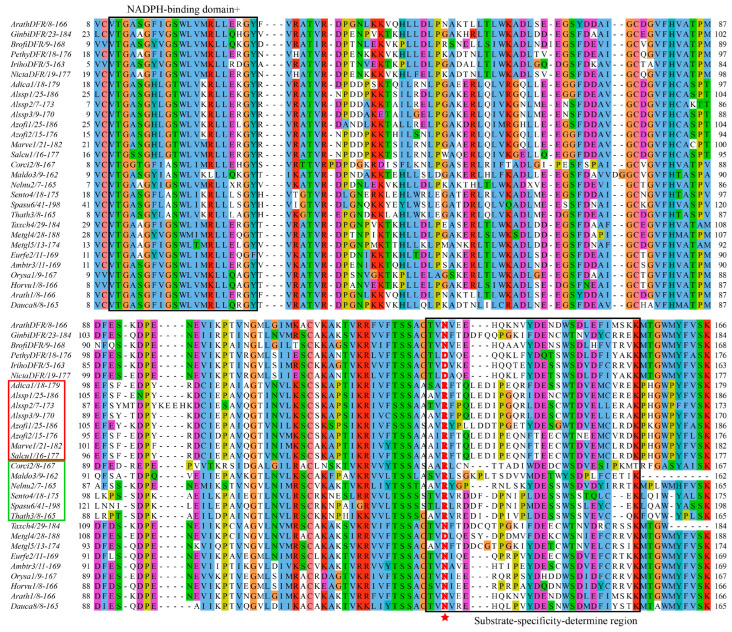
Multiple alignment analysis of the amino acid sequences of putative DFRs. The putative NAD(P)-binding domain and the putative substrate-binding domain are circled with two black boxes. The Arg-type DFR of the ferns are circled with red boxes. The Arg-type DFR of the eudicots is circled with green boxes. Red asterisk indicates amino acid residue 134, which is particularly important for substrate recognition. Plotted using Jalview (v2.11.4.1).

Previous studies have identified four types of DFRs (Asn-, Asp-, Arg-, and non-Asp/Asn/Arg-types) based on the amino acid residue at position 134, a key site within the substrate-specificity-determining region [22,46]. Specifically, DFRs with an asparagine (N) at this position are designated as Asn-type, those with aspartic acid (D) as Asp-type, and those with arginine (R) as Arg-type. DFRs containing amino acids other than Asp, Asn, or Arg at this position are classified as the non-Asp/Asn/Arg-type. To examine the types of 745 DFR protein sequences, we downloaded six functionally validated plant DFR protein sequences from the National Center for Biotechnology Information (NCBI) database (accession numbers of the DFR sequences; see Section 2). Using these six sequences as references, we performed multiple sequence alignment of the 745 DFR sequences with MAFFT and visualized the results using Jalview software. By analyzing the amino acid residues within the DFR substrate-binding region, we identified 207 sequences with asparagine at position 134, 70 sequences with aspartic acid, and 14 sequences with arginine (Figure S1 and Figure 2, Table S3). These sequences were classified as Asn-type, Asp-type, and Arg-type, respectively. Additionally, sequences with amino acid residues other than asparagine, aspartic, and arginine at position 134 were classified as non-Asn/Asp/Arg-types (Table S3). The distributions of these four DFR types varied significantly among the different plant taxa. Asn- and Asp-type DFRs are restricted to seed plants (gymnosperms, basal angiosperms, and angiosperms). Arg-type DFRs are found in ferns and some dicotyledonous plants, and non-Asn/Asp/Arg-type DFRs are widespread in all taxa, from ferns to angiosperms. Interestingly, among the nine DFR sequences identified in ferns, eight were classified as Arg-type and one was classified as non-Asn/Asp/Arg-type.

### 3.2. Phylogenetic Analysis of DFR Gene Family

**Figure 3:**
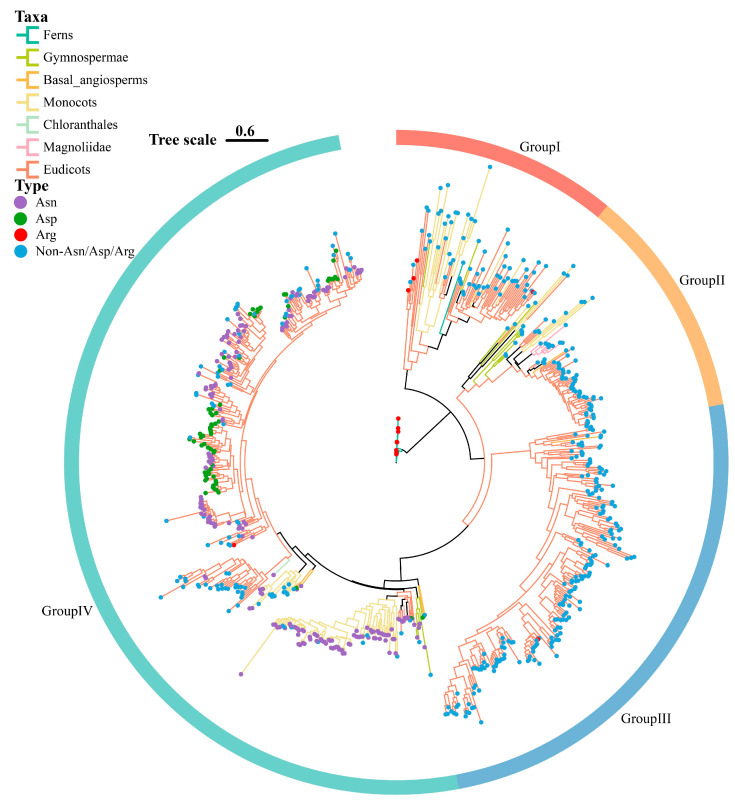
The phylogenetic tree of *DFR* gene family. The seven colors of the phylogenetic tree branches represent different plant taxa, and the four colored dots on the branches represent the four types of DFRs. Plotted using ChiPlot.

To explore the phylogenetic relationships among *DFR* gene families across different plant taxa, we constructed a phylogenetic tree of *DFR* gene families, with ferns as outgroups (Figure 3). Based on topology, the DFR protein sequences were divided into Groups I, II, III, and IV (Figure 3). The results showed that the DFR sequences of the ferns were clustered into Group I. The DFR sequences of gymnosperms and monocots were clustered into Groups I, II, and III. The DFR sequences of the basal angiosperms and eudicots were clustered into all four groups. The DFR sequences of chloranthales were clustered into Group IV. The DFR sequences of magnoliids were clustered into Groups II, III, and IV. Overall, most branches of all four groups contained DFR sequences from the various plant taxa. Notably, DFR sequences from early diverging species were located at the base of the branches in the phylogenetic tree, suggesting that these DFR sequences in each branch evolved independently following species divergence.

The four types of DFR protein sequences were mapped onto a phylogenetic tree, revealing a dispersed distribution pattern across all groups (Figure 3). The non-Asn/Asp/Arg-type was broadly distributed among the four groups, whereas the Arg-type was predominantly localized within Group I, with a smaller distribution in Groups III and IV. Remarkably, Asn- and Asp-types were exclusively present in Group IV. To further confirm the distribution of Asn- and Asp-types, we downloaded 22 DFR protein sequences from NCBI that included these two types (Table S4) and aligned them with the six reference sequences and 745 DFR protein sequences, subsequently reconstructing the phylogenetic tree comprising a total of 773 DFR sequences (Supplemental Figure S2). Remarkably, all 28 DFR protein sequences of the known functional types were clustered in Group IV.

### 3.3. Member Number and Distribution of the DFR Gene Family Across Different Plant Taxa

**Figure 4:**
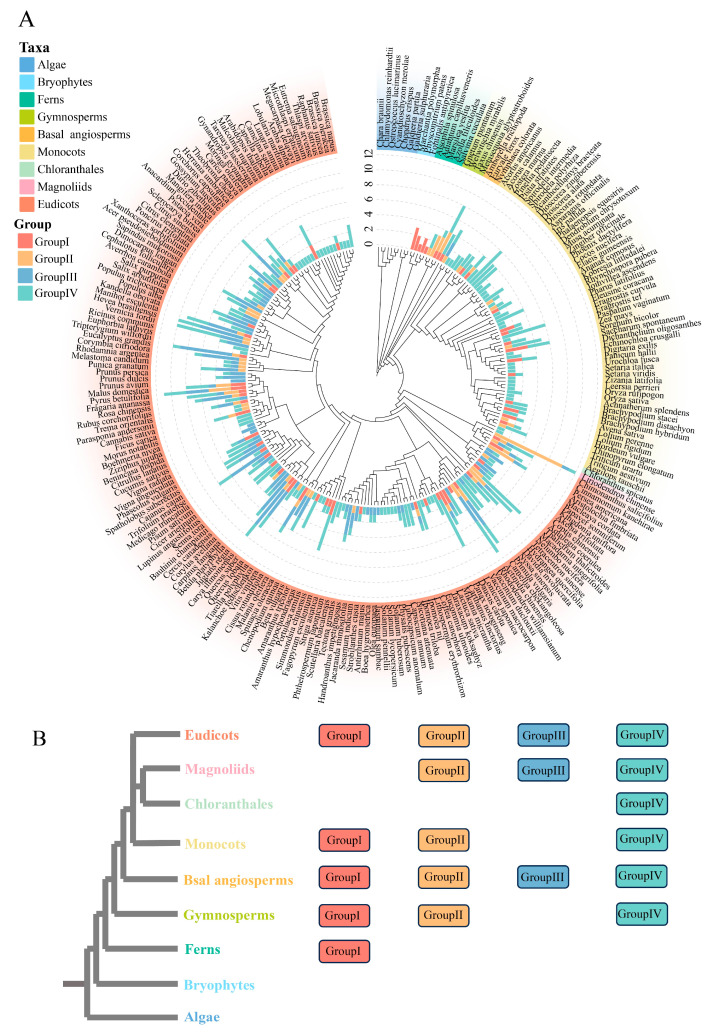
The distribution of *DFR* gene families across species. (**A**) The number of *DFR* gene family members in 237 plant species. Nine major plant lineages are represented by different colors; the total length of the bar in the middle indicates the *DFR* member number of each species; and the four colors of the bar species indicate the number of *DFR* members in each species in each of the four topologies. (**B**) Distribution of each *DFR* gene family topology in different plant taxa. The colors in 9 indicate different plant lineages, and the solid round rectangles indicate the gene topologies of the *DFR* family present in the corresponding plant lineages. Plotted using ChiPlot.

We counted the number of *DFR* family members and their distributions in different plant taxa, which were presented in a phylogenetic tree for 237 plant species (Figure 4A and Figure S3, Table S2). Our analyses revealed that *DFR* family members are distributed in five ferns, suggesting that the origin of *DFR* families can be traced back to the fern period. We did not identify *DFR* members in algae and mosses, and in ferns, DFR usually contains 1 to 3 members. In gymnosperms, the member number increases from 3 to 8, while in angiosperms, the number of *DFR* family members varies widely, ranging from 1 to 12. This variation in member number may be linked to environmental changes and the evolving need for flavonoid pigments in different plant lineages.

Additionally, the distribution of each DFR sequence topology across plant taxa was mapped by combining the phylogenetic tree and species relationships (Figure 4B). These four groups were absent in algae and bryophytes. Group I first appeared in ferns but is missing in certain angiosperms, such as chloranthales and magnoliids. Groups II and IV first occurred in gymnosperms, but Group IV continued to develop into all lineages of angiosperms, whereas Group II was lost in the Chloranthales lineage of angiosperms. Group III, which was first detected in basal angiosperms, is missing from the monocots and chloranthales lineages.

### 3.4. Conserved Motif Analysis of Representative Species

To analyze the conserved motifs, we selected 25 representative plant species spanning various plant taxa, including: the ferns *Alsophila spinulosa* (*A. spinulosa*), *Azolla filiculoides* (*A. filiculoides*), and *Salvinia cucullate* (*S. cucullate*); the gymnosperms *Gnetum montanum* (*G. montanum*), *Welwitschia mirabilis* (*W. mirabilis*), and *Taxus chinensis* (*T. chinensis*); the basal angiosperms *Nymphaea colorata* (*N. colorata*), *Amborella trichopoda* (*A. trichopoda*), and *Euryale ferox* (*E. ferox*); the monocots *Avena sativa* (*A. sativa*), *Brachypodium distachyon* (*B. distachyon*), *Dioscorea alata* (*D. alata*), *Musa acuminata* (*M. acuminata*), *O. sativa*, *Setaria viridis* (*S. viridis*), *Triticum aestivum* (*T. aestivum*); the chloranthale *Chloranthus spicatus* (*C. spicatus*), the magnoliidae *Liriodendron chinense* (*L. chinense*); the eudicots *A. thaliana*, *B. napus*, *Citrus clementina* (*C. clementina*), *Citrus sinensis* (*C. sinensis*), *Medicago truncatula* (*M. truncatula*), *Prunus persica* (*P. persica*), *Solanum lycopersicum* (*S. lycopersicum*).

**Figure 5:**
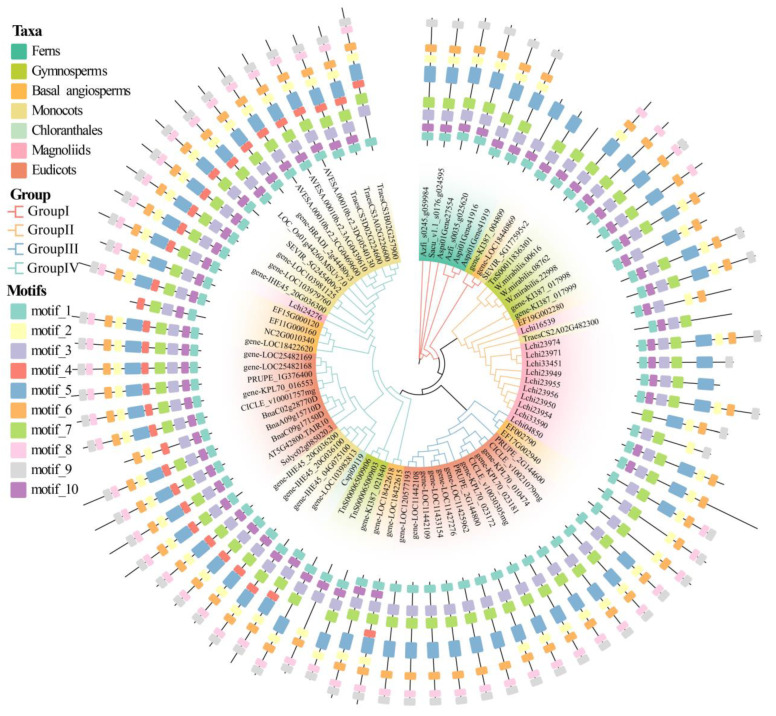
The phylogenetic and conserved motif analysis of 25 representative species. The four branch colors in the phylogenetic tree represent four groups, and the seven colors of the gene IDs indicate different plant taxa. The outermost circle shows the distribution of 10 motifs. Plotted using ChiPlot.

We conducted a phylogenetic analysis of 80 DFR protein sequences from 25 species and investigated the conserved motifs within the *DFR* gene family utilizing the MEME software. The maximum number of motifs was set to 10 (Figure 5 and Figure S4). Motif 1 (8–29 aa in *AT5G42800.TAIR10* labeling) corresponded to the NAD(P)H-binding domain, Motifs 2–10 did not match any functional annotation. Of the 80 DFRs, nearly all proteins contained Motifs 1, 3, 5, and 7, indicating that these motifs are highly conserved. In contrast, Motifs 2, 4, 6, and 8–10 exhibited partial absence across the four phylogenetic groups. Interestingly, the absence of Motifs 4, 8, and 10 varied markedly among groups. For example, Motif 4 was detected only in angiosperms within Group IV, suggesting that Group IV sequences underwent further functional differentiation in angiosperms. Motif 8 was absent only in Group I, which may indicate that this motif emerged after Group I diverged from the others, whereas Motif 10 was missing only in Group III, indicating that it was lost during the evolution of that group.

### 3.5. Identification of Gene Duplication Types

**Figure 6:**
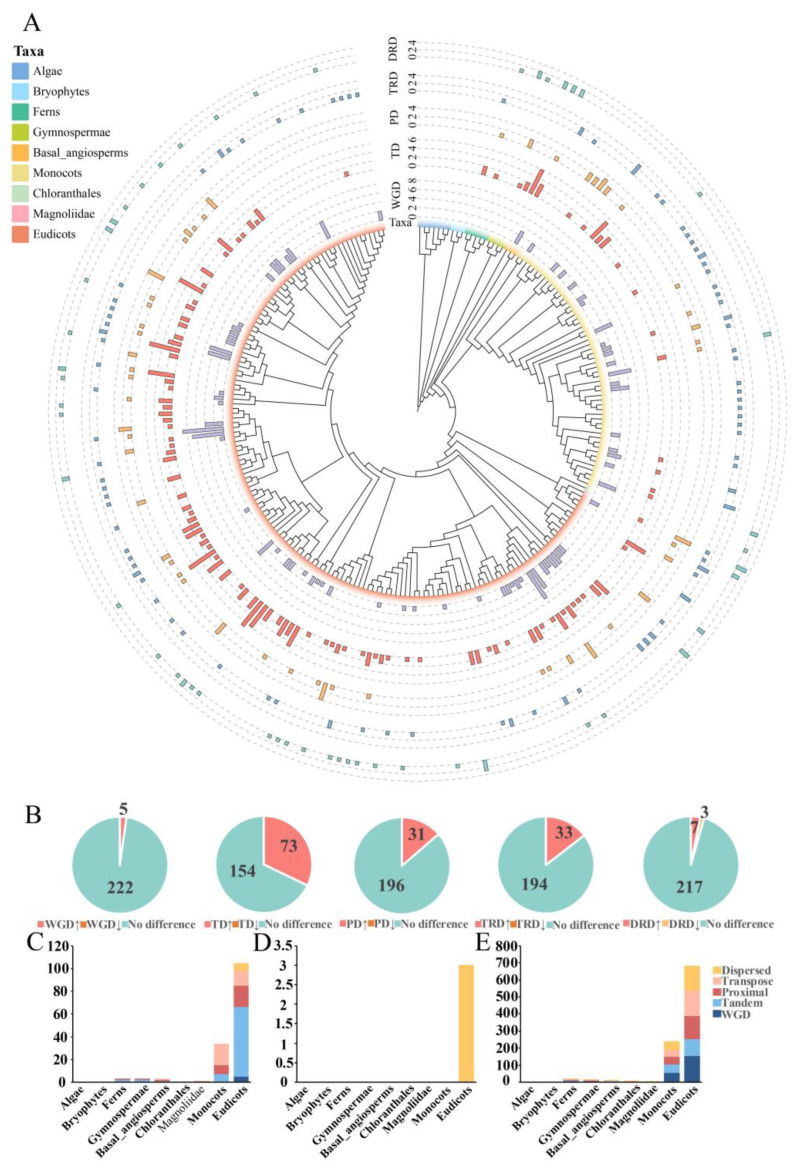
The analysis of *DFR* gene family duplication types. (**A**) Display of the number of each replication type of *DFR* family genes for 237 plant species. From the inside out are the species phylogenetic tree, number of WGD types of *DFR* family genes in each species, number of TD types of *DFR* family genes in each species, number of PD types of *DFR* family genes in each species, number of TRD types of *DFR* family genes in each species, and number of DRD types of *DFR* family genes in each species. (**B**) The number of significant increases or decreases and insignificant changes in each repeat type of *DFR* family genes. Significant increases and significant decreases are represented by upward and downward arrows, respectively. (**C**) Number of species with significant increases in repeat types for each taxonomic unit of *DFR* family genes. (**D**) Number of species with significant decreases in repeat type per taxonomic unit for *DFR* family genes. (**E**) Number of species with non-significant changes in repeat type for each taxonomic unit of *DFR* family genes. Plotted using ChiPlot.

To explore the duplication history of the *DFR* gene family in plants, we counted the distribution of the *DFR* family across these six duplication types in each species (Figure 6A and Figure S5, Tables S5–S10). The results revealed that the SL type was absent in all species, while the remaining five duplication types were found across different species. Analysis indicated that WGD and TD were the predominant duplication types for *DFR* family genes. WGD events were particularly prominent in species such as *Camelina sativa* (*C. sativa*), *P. betulifolia*, *Saccharum spontaneum* (*S. spontaneum*), and *Bauhinia championii* (*B. championii*), suggesting that polyploidization events may play a key role in the amplification of the *DFR* gene family in these plants, as well as contributing to the regulation and diversification of anthocyanin biosynthesis pathways. On the other hand, TD were dominant across nearly all taxa, implying their crucial role in maintaining and expanding the *DFR* gene family.

Furthermore, we performed chi-square tests to compare the differences in the numbers of the five duplication types at both the gene family and genomic levels, further exploring the specific contributions of each duplication type to the expansion of the *DFR* gene family. (Figure 6B, Tables S5–S10). For the WGD type (Figure 6B, Table S5), compared to the number of WGD at the genomic level, five species were significantly enriched for WGD in the *DFR* family genes, while none had a significantly lower number of WGD of the *DFR* family genes. For the TD type (Figure 6B, Table S6), compared to the number of TD at the genomic level, 73 species were significantly enriched for TD in the *DFR* family genes, while none had a significantly lower number of TD in the *DFR* family genes. For the PD type (Figure 6B, Table S7), compared to the number of PD at the genomic level, 31 species were significantly enriched for PD in the *DFR* family genes, while none had a significantly lower number of PD in the *DFR* family genes. For the TRD type (Figure 6B, Table S8), compared to the number of TRD at the genomic level, 33 species were significantly enriched for TRD in the *DFR* family genes, while none had a significantly lower number of TRD in the *DFR* family genes. For the DRD type (Figure 6B, Table S9), compared to the number of DRD at the genomic level, seven species were significantly enriched for DRD in the *DFR* family genes. A total of three species had significantly lower DRD in the *DFR* family genes. These results suggest that TD and TRD are the primary mechanisms driving *DFR* family gene expansion across most species. We further explored the distribution of duplicate types in terms of each plant taxon, with the results presented in Figure 6C–E. There were no significant differences in expansion mechanisms between lower and higher plants. Ferns, gymnosperms, basal angiosperms, magnoliidae, and eudicots primarily expanded the *DFR* family through TD and PD, while monocots mainly underwent expansion through PD and TD.

### 3.6. Ka/Ks Calculation

**Figure 7:**
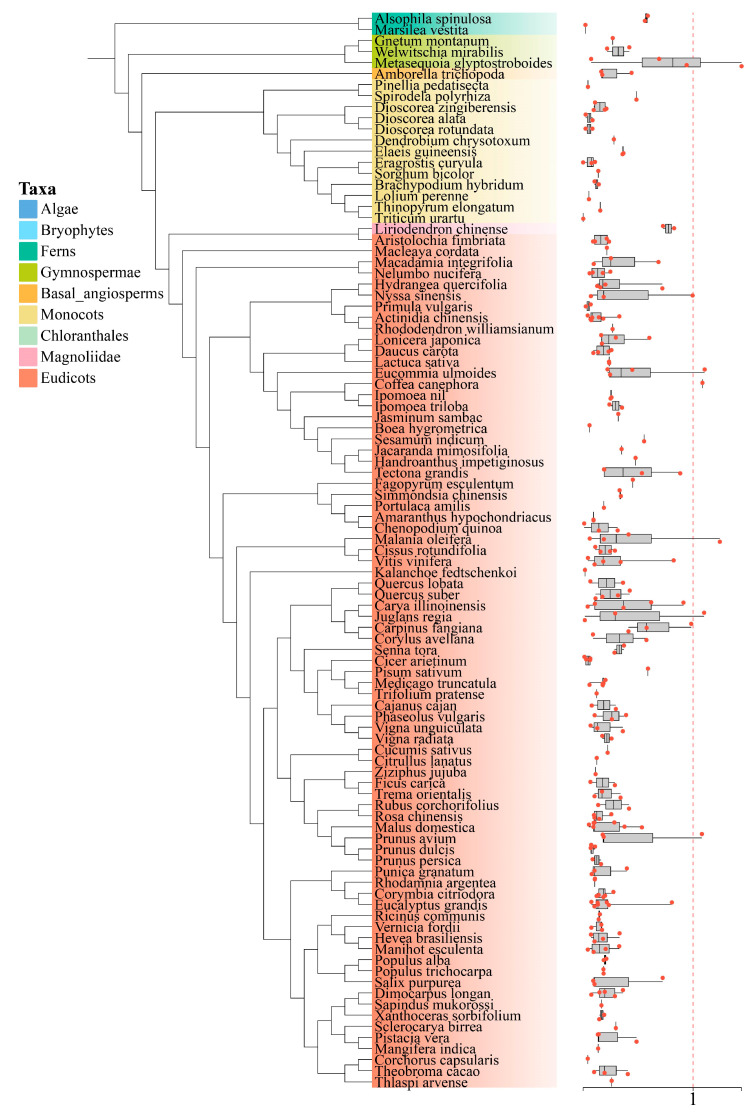
The analysis of ka/ks values for *DFR* family homologous gene pairs. The seven colors of species names on the phylogenetic tree represent different plant taxa, and the box plots represent the distribution of Ka/Ks values. A point on the box plot represents a ka/ks value. Plotted using ChiPlot.

To assess the selection pressures acting on the *DFR* gene family, we focused on two major duplication types and calculated the nonsynonymous (Ka) and synonymous (Ks) substitution rates for selected paralogous gene pairs. The Ka/Ks ratios were computed, and the results were visualized as a box plot (Figure 7). The analysis revealed that most paralogous gene pairs had a Ka/Ks ratio < 1, indicating that these genes underwent purifying selection. Some gene pairs lacked Ka/Ks values, suggesting substantial sequence divergence. Interestingly, only a small number of the homologous gene pairs exhibited a Ka/Ks ratio > 1 (Tables S11 and S12), implying that, while most *DFR* genes were under purifying selection and maintained functional conservation, a small proportion may have undergone functional divergence or adaptive changes during evolution.

### 3.7. Expression Analysis of DFR Genes Under Different Conditions

**Figure 8:**
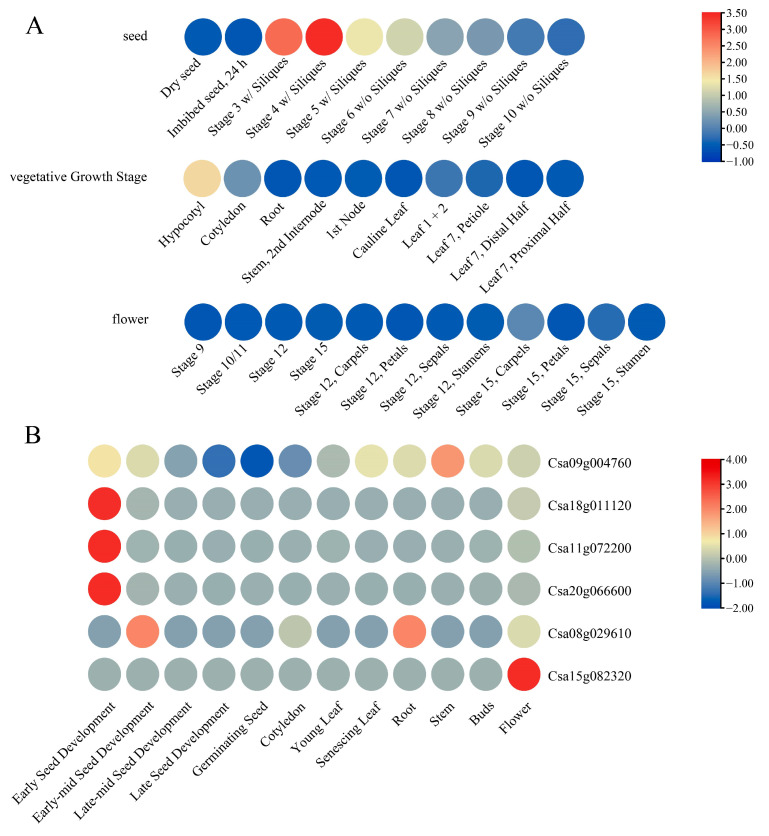
Expression analysis of *DFR* family members in *Arabidopsis thaliana* (*A. thaliana*) and *Camelina sativa* (*C. sativa*). (**A**) The expression levels of *DFR* family genes in *A. thaliana* under different stages of development and tissues. (**B**) The expression of *DFR* family genes in *C. sativa* across different stages of development and tissues. Plotted using TBtools (v2.119).

To explore the expression patterns of *DFR* family genes, we collected expression data from *A. thaliana* and *C. sativa* at different developmental stages and in various tissues (Figure 8, Tables S13 and S14). With respect to the expression of the *DFR* gene (*AT5G42800*) *A. thaliana* (Figure 8A, Table S13), we observed that this Asn-type *DFR* gene was significantly upregulated during the early stages of seed development.

With respect to the expression of the *DFR* genes in *C. sativa* (Figure 8B, Table S14), we found significant differences in expression among these *DFR* genes. Three Asn-type DFR genes (*Csa20G066600*, *Csa18g011120*, and *Csa11g072200*) exhibited high expression levels during early seed development. *Csa15g082320* showed high expression primarily in flowers. *Csa08g029610* was highly expressed during the early-to-mid-seed development and in roots, while *Csa09g004760* had significant expression in stems. These results suggest that different *DFR* genes in *C. sativa* may have different roles in different tissues.

Notably, the Asn-type *DFR* genes in both *A. thaliana* and *C. sativa* were highly expressed during early seed development.

## 4. Discussion

The production of anthocyanins is a critical adaptive response by plants to environmental stress. As a crucial branchpoint enzyme in the biosynthesis of anthocyanin precursors, dihydroflavonol 4-reductase (DFR) plays an indispensable role in plant stress adaptation. For example, in purple sweet potato [47], expression of the *IbDFR* gene was strongly associated with anthocyanin accumulation in leaves, stems, and roots, while downregulation of *IbDFR* expression significantly reduces both anthocyanin levels and antioxidant capacity. Similarly, in *Brassica napus* (*B. napus*) [48], overexpression of *Arabidopsis DFR* in *B. napus* not only elevates anthocyanin content but also reduces the accumulation of reactive oxygen species (ROS) and enhances salt tolerance. Moreover, studies on different varieties of foxtail millet have shown that varieties with more anthocyanin accumulation and stronger stress tolerance exhibit significantly higher expression levels of *DFR* genes [49]. Collectively, these examples provide strong evidence that DFR-mediated anthocyanin synthesis plays a pivotal role in enhancing plant resistance to environmental stresses. The current investigations of the *DFR* gene family are based on single or limited species, lacking comprehensive systematic identification and analysis in the plant kingdom. Our large-scale *DFR* gene family analysis provides novel insights into the evolutionary trajectory and functional diversification mechanisms.

In this study, we identified 745 DFR protein sequences from 237 plant species, and the absence of DFR homologs in algae and bryophytes, coupled with their conserved presence in ferns and seed plants. In current studies, *DFR* genes have not been found in algae and mosses. Therefore, we hypothesized that the *DFR* gene family may have originated from the common ancestor of ferns and seed plants. This coincides with key innovations during plant terrestrialization, suggesting that DFR-mediated flavonoid diversification may be indispensable for overcoming abiotic stresses such as UV radiation and desiccation. Notably, as plants transitioned from aquatic to terrestrial environments, the member number of the *DFR* gene family gradually increased. Terrestrial plants face many environmental stresses that aquatic plants do not encounter [50]. Since early land plants lacked seeds and flowers, the function of pigments flavonoid (anthocyanins) was likely unrelated to interactions with animals [51]. The emergence of DFR—from algae to bryophytes and then to vascular plants (ferns)—may be linked to the need to adapt to a range of abiotic stresses, such as strong light, drought, and ultraviolet radiation. In seed plants (gymnosperms, angiosperms), the member number of the *DFR* gene family increases significantly, a change that may be associated with the complex plant-animal interactions [51]. For example, the higher *DFR* gene family number in angiosperms may reflect an enhancement of anthocyanin-mediated visual signaling for frugivore attraction [52], which in turn aids in seed dispersal. Such adaptations likely enabled angiosperms to exploit novel ecological niches through co-evolutionary relationships with animal dispersers.

In previous studies on the *DFR* gene family in *B. napus* [26], Solanaceae [27], and tea [25], *DFR* genes were consistently classified into three types (Asn, Asp, and non-Asn/Asp). In this study, based on recent research on the Arg-type DFR in the fern (*D. erythrosora*) and the critical residue variations in the substrate-specific regions, we further classified the *DFR* gene family into four types: Asn-type, Asp-type, Arg-type, and non-Asn/Asp/Arg-type. Among these 745 DFR protein sequences, we identified 207 Asn-type, 70 Asp-type, 14 Arg-type, and 454 non-Asn/Asp/Arg-type sequences. Notably, the Asn-and Asp-type identification for *Hordeum vulgare* (*H. vulgare*) [53], *Daucus carota* (*D. carota*) [54], *Medicago truncatula* (*M. truncatula*) [55], *Solanum tuberosum* (*S. tuberosum*) [56], and *S. lycopersicum* [27] aligned with previous reports. The Arg-type DFRs, previously considered fern-specific, were unexpectedly detected in several eudicots. Conversely, Asn-type and Asp-type DFRs appeared exclusively in seed plants, with their dual presence in gymnosperms like *Metasequoia glyptostroboides* and *Ginkgo biloba* (*G. biloba*) [57] suggesting their divergence predates the gymnosperms radiation. Interestingly, Asn-type DFRs were exclusively located in phylogenetic Group IV, a clade unique to seed plants, and exhibited marked upregulation during early seed developmental stages (e.g., *A. thaliana* and *C. sativa*). It is, therefore, implied that Group IV likely played a pivotal role in the terrestrial adaptation of seed plants.

Different forms of gene duplications play a crucial role in plant evolution [58]. Duplicated genes are retained in evolutionary processes through subfunctionalization and neofunctionalization [59]. Our large-scale identification and analysis of gene duplication types in the *DFR* gene family revealed that TD was the major duplication type in the *DFR* gene family. Further chi-square test analysis indicated that TD is also a major mechanism driving the expansion of the *DFR* gene family. Analyses of apple *DFR* genes preliminarily suggested that TD might have been the major reason for its expansion [28]. Meanwhile, research on azalea genomes by Yang et al. found that TD/PD in azaleas had substantially contributed to the proportions of enzymatic genes for the anthocyanin biosynthesis pathway [60]. Collectively, these results underscore the critical role of TD in the expansion of the *DFR* gene family. TD introduces novel gene copies into the genome, thereby providing the potential for the evolution of novel functional genes [61,62]. Based on these findings, we believe that TD events, as the predominant driver of *DFR* family expansion, may be intrinsically linked to the emergence of four types of DFR (Asn, Asp, Arg, and non-Asn/Asp/Arg).

Flavonoid biosynthesis branches off from the general phenylpropanoid pathway, with chalcone synthase (CHS) and chalcone isomerase (CHI) serving as the first and second committed enzymes, respectively, and playing a critical role in the entire flavonoid pathway [63,64]. Studies have shown that the origin of the *CHS* gene family can be traced back to the algal period [65], significantly earlier than that of the *DFR* gene family. As the first enzyme in flavonoid biosynthesis, the *CHS* gene family played a key role during the early adaptation of plants transitioning from aquatic to terrestrial environments, whereas the *DFR* gene family likely evolved gradually during later stages of terrestrial adaptation and diversification. As members of the CHI-folding protein superfamily, CHIs similarly include four subclasses: Type I CHIs are widely present in vascular plants [66]; Type II CHIs were once considered specific to legumes [67], but have also been identified in ancient land plants, including liverworts and Selaginella [68]; in contrast, Type III CHIs are widely distributed in both land plants and green algae [69], while Type IV CHIs are thought to be restricted to land plants [69]. However, the distribution patterns of these two gene families are markedly different. This divergence in distribution may reflect the distinct adaptive demands that plants have faced at various stages of evolution. Specifically, the diversification of the *CHI* gene family likely mirrors early evolutionary events associated with the establishment of the flavonoid pathway, whereas the diversification of the *DFR* gene family appears to be closely linked to later adaptive events, particularly during the diversification of seed plants and the evolution of their reproductive strategies. In conclusion, this study provides valuable insights that will inform future research on the *DFR* gene family in other species.

## Data Availability

The original contributions presented in the study are included in the Supplementary Materials. Further inquiries can be directed to the corresponding authors.

## Supplementary Materials

The following supporting information can be downloaded at: www.mdpi.com/article/10.3390/genes16040396/s1. Figure S1. The results of the multiple sequence comparison of 277 Asn- and Asp-type DFR; Figure S2. The distribution of 28 reference DFRs on the phylogenetic tree; Figure S3. The phylogenetic relationships of 237 plant species; Figure S4. The composition of conserved motifs of the *DFR* genes; Figure S5. The heatmap of the percentage of *DFR* family gene duplication types in each species; Table S1. The genome download sources for 237 species; Table S2. The *DFR* Gene Family Counts in 237 Species: Total and Distribution Across Topological Structures; Table S3. The 745 DFR Protein Sequences Information; Table S4. The DFR sequences with known functional types; Table S5. The statistics and significance analysis of the number of *DFR* WGD duplication genes in 237 species with *p*-value < 0.05; Table S6. The statistics and significance analysis of the number of *DFR* tandem duplication genes in 237 species with *p*-value < 0.05; Table S7. The statistics and significance analysis of the number of *DFR* proximal duplication genes in 237 species with *p*-value < 0.05; Table S8. The statistics and significance analysis of the number of *DFR* transpose duplication genes in 237 species with *p*-value < 0.05; Table S9. The statistics and significance analysis of the number of *DFR* dispersed duplication genes in 237 species with *p*-value < 0.05; Table S10. The statistics and significance analysis of the number of *DFR* singletons duplication genes in 237species with *p*-value < 0.05; Table S11. The distribution of paralogous with ka/ks in WGD-type DFR genes in 237 species; Table S12. The distribution of paralogous with ka/ks in tandem-type *DFR* genes in 237 species; Table S13. The absolute expression values of *DFR* family genes in *A. thaliana* during various developmental stages in different tissues obtained from the *Arabidopsis* eFP Browser; Table S14. The absolute expression values of *DFR* family genes in *Camelina sativa* during various developmental stages in different tissues obtained from the *C. sativa* eFP Browser; Python script: filter_busco_result_80: Extraction of Single-Copy Gene Sequences With More Than 80% Coverage; S2. species_gene_family_member_statistics: Statistics of the Number of DFR Sequences in Each Species; S3. gene_duplication_type_statistics1: Statistics of Duplication Types in Each Species and the DFR Gene Family: Step One; S4. gene_duplication_type_statistics2: Statistics of Duplication Types in Each Species and the DFR Gene Family: Step Two.
