# Supplementary material for: A Comprehensive Evolutionary Analysis of the Dihydroflavonol 4-Reductase (DFR) Gene Family in Plants: Insights from 237 Species
Source: Genes (Basel). 2025 Mar 29;16(4):396. doi: 10.3390/genes16040396 (PMC12027299; doi:10.3390/genes16040396)
Supplement: Supplementary file 1 [file genes-16-00396-s001.zip › supplementary material/Figure S3.pdf]

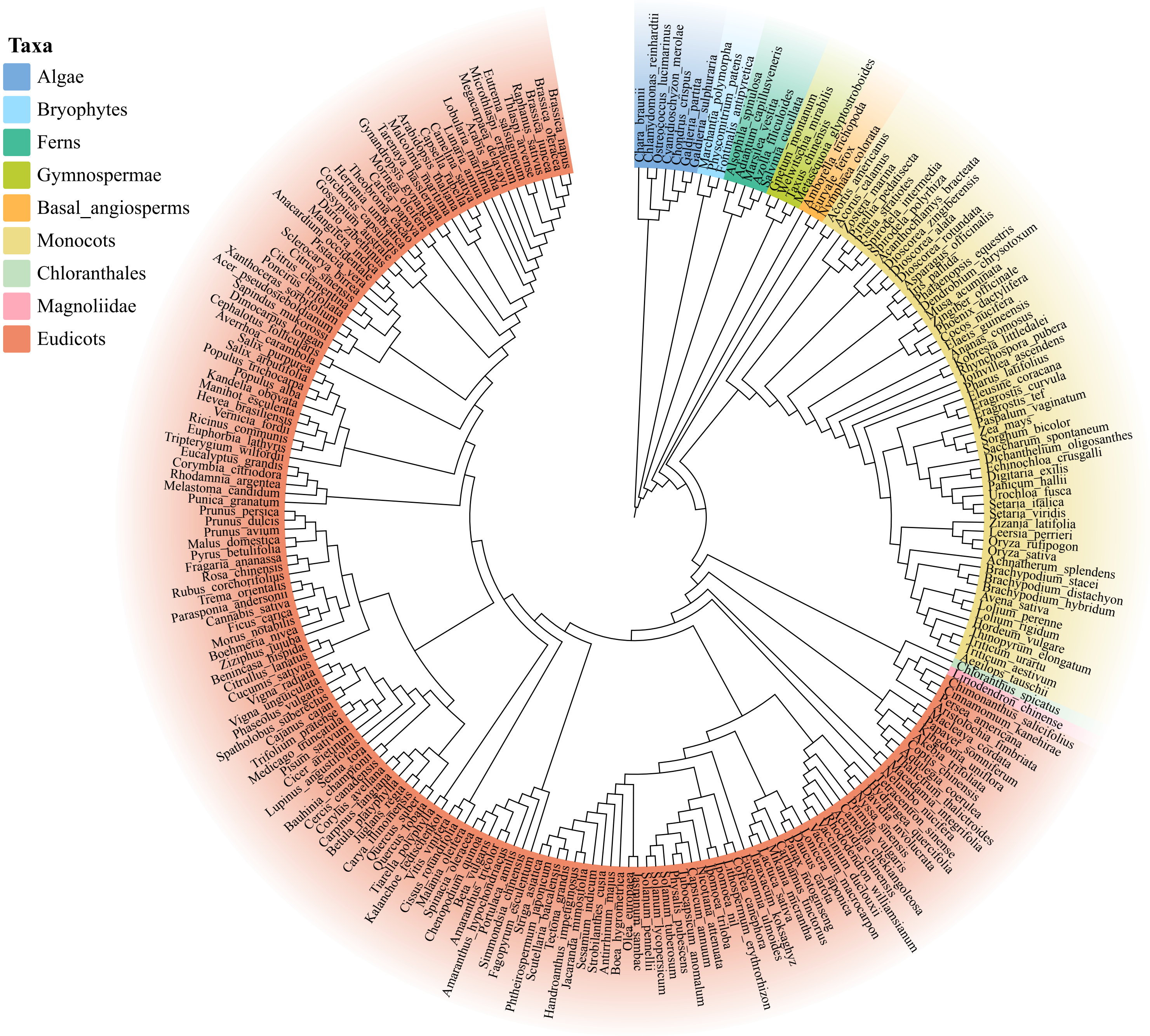
Taxa
Algae
Bryophytes
Ferns
Gymnospermae
Basal_angiosperms
Monocots
Chloranthales
Magnoliidae
Eudicots
Chara_braunii
Chlamydomonas_reinhardtii
Ostreococcus_lucimarinus
Cyanidioschyzon_merolae
Chondrus_crispus
Galdieria_partita
Galdieria_sulphuraria
Marchantia_polymorpha
Physcomitrium_patens
Fontinalis_antipyretica
Alsophila_spinulosa
Adiantum_capillusveneris
Marsilea_vestita
Azolla_filiculoides
Salvinia_cucullata
Gnetum_montanum
Welwitschia_mirabilis
Taxus_chinensis
Metasequoia_glyptostroboides
Amborella_trichopoda
Euryale_ferox
Nymphaea_colorata
Acorus_americanus
Acorus_calamus
Zostera_marina
Pinellia_pedatisecta
Pistia_stratiotes
Spirodela_intermedia
Spirodela_polyrhiza
Acanthochlamys_bracteata
Dioscorea_zingiberensis
Dioscorea_alata
Dioscorea_rotundata
Asparagus_officinalis
Iris_pallida
Phalaenopsis_equestris
Dendrobium_chrysotoxum
Musa_acuminata
Zingiber_officinale
Phoenix_dactylifera
Cocos_nucifera
Elaeis_guineensis
Ananas_comosus
Kobresia_littledalei
Rhynchospora_pubera
Joinvillea_ascendens
Pharus_latifolius
Eleusine_coracana
Eragrostis_curvula
Eragrostis_tef
Paspalum_vaginatum
Zea_mays
Sorghum_bicolor
Saccharum_spontaneum
Dichanthelium_oligosanthes
Echinochloa_crusgalli
Digitaria_exilis
Panicum_hallii
Urochloa_fusca
Setaria_italica
Setaria_viridis
Zizania_latifolia
Leersia_perrieri
Oryza_rufipogon
Oryza_sativa
Achnatherum_splendens
Brachypodium_stacei
Brachypodium_distachyon
Brachypodium_hybridum
Avena_sativa
Lolium_perenne
Lolium_rigidum
Hordeum_vulgare
Thinopyrum_elongatum
Triticum_urartu
Triticum_aestivum
Aegilops_tauschii
Chloranthus_spicatus
Liriodendron_chinense
Chimonanthus_salicifolius
Cinnamomum_kanehirae
Persea_americana
Aristolochia_fimbriata
Macleaya_cordata
Papaver_somniferum
Kingdonia_uniflora
Akebia_trifoliata
Coptis_chinensis
Aquilegia_coerulea
Thalictrum_thalictroides
Macadamia_integrifolia
Nelumbo_nucifera
Tetracentron_sinense
Hydrangea_quercifolia
Davidia_involucrata
Nyssa_sinensis
Primula_vulgaris
Camellia_chekiangoleosa
Actinidia_chinensis
Rhododendron_williamsianum
Vaccinium_macrocarpon
Vaccinium_duclouxii
Lonicera_japonica
Daucus_carota
Panax_notoginseng
Carthamus_tinctorius
Mikania_micrantha
Lactuca_sativa
Taraxacum_koksaghyz
Eucommia_ulmoides
Coffea_canephora
Lithospermum_erythrorhizon
Ipomoea_nil
Ipomoea_triloba
Nicotiana_attenuata
Capsicum_annuum
Tubocapsicum_anomalum
Physalis_pubescens
Solanum_tuberosum
Solanum_lycopersicum
Solanum_pennellii
Jasminum_sambac
Olea_europaea
Boea_hygrometrica
Antirrhinum_majus
Strobilanthes_cusia
Sesamum_indicum
Jacaranda_mimosifolia
Handroanthus_impetiginosus
Tectona_grandis
Scutellaria_baicalensis
Phtheirospermum_japonicum
Striga_asiatica
Fagopyrum_esculentum
Simmondsia_chinensis
Portulaca_amilis
Amaranthus_hypochondriacus
Amaranthus_tricolor
Beta_vulgaris
Chenopodium_quinoa
Spinacia_oleracea
Malania_oleifera
Cissus_rotundifolia
Vitis_vinifera
Kalanchoe_fedtschenkoi
Tiarella_polyphylla
Quercus_lobata
Quercus_suber
Carya_illinoinensis
Juglans_regia
Betula_platyphylla
Carpinus_fangiana
Corylus_avellana
Cercis_canadensis
Bauhinia_championii
Senna_tora
Lupinus_angustifolius
Cicer_arietinum
Pisum_sativum
Medicago_truncatula
Trifolium_pratense
Cajanus_cajan
Spatholobus_suberectus
Phaseolus_vulgaris
Vigna_unguiculata
Vigna_radiata
Cucumis_sativus
Citrullus_lanatus
Benincasa_hispida
Ziziphus_jujuba
Boehmeria_nivea
Morus_notabilis
Ficus_carica
Cannabis_sativa
Parasponia_andersonii
Trema_orientalis
Rubus_corchorifolius
Rosa_chinensis
Fragaria_ananassa
Pyrus_betulifolia
Malus_domestica
Prunus_avium
Prunus_dulcis
Prunus_persica
Punica_granatum
Melastoma_candidum
Rhodamnia_argentea
Corymbia_citriodora
Eucalyptus_grandis
Tripterygium_wilfordii
Euphorbia_lathyris
Ricinus_communis
Vernicia_fordii
Hevea_brasiliensis
Manihot_esculenta
Kandelia_obovata
Populus_alba
Populus_trichocarpa
Salix_arbutifolia
Salix_purpurea
Averrhoa_carambola
Cephalotus_follicularis
Dimocarpus_longan
Sapindus_mukorossi
Acer_pseudosieboldianum
Xanthoceras_sorbifolium
Poncirus_trifoliata
Citrus_clementina
Citrus_sinensis
Sclerocarya_birrea
Pistacia_vera
Anacardium_occidentale
Mangifera_indica
Durio_zibethinus
Gossypium_australe
Corchorus_capsularis
Herrania_umbratica
Theobroma_cacao
Carica_papaya
Moringa_oleifera
Gynandropsis_gynandra
Tarenaya_hassleriana
Malcolmia_maritima
Arabidopsis_thaliana
Capsella_rubella
Camelina_sativa
Lunaria_annua
Lobularia_maritima
Arabis_alpina
Megacarpaea_delavayi
Microthlaspi_erraticum
Eutrema_salsugineum
Thlaspi_arvense
Raphanus_sativus
Brassica_juncea
Brassica_oleracea
Brassica_napus
