# Supplementary material for: A Comprehensive Evolutionary Analysis of the Dihydroflavonol 4-Reductase (DFR) Gene Family in Plants: Insights from 237 Species
Source: Genes (Basel). 2025 Mar 29;16(4):396. doi: 10.3390/genes16040396 (PMC12027299; doi:10.3390/genes16040396)
Supplement: Supplementary file 1 [file genes-16-00396-s001.zip › supplementary material/Figure S5.pdf]

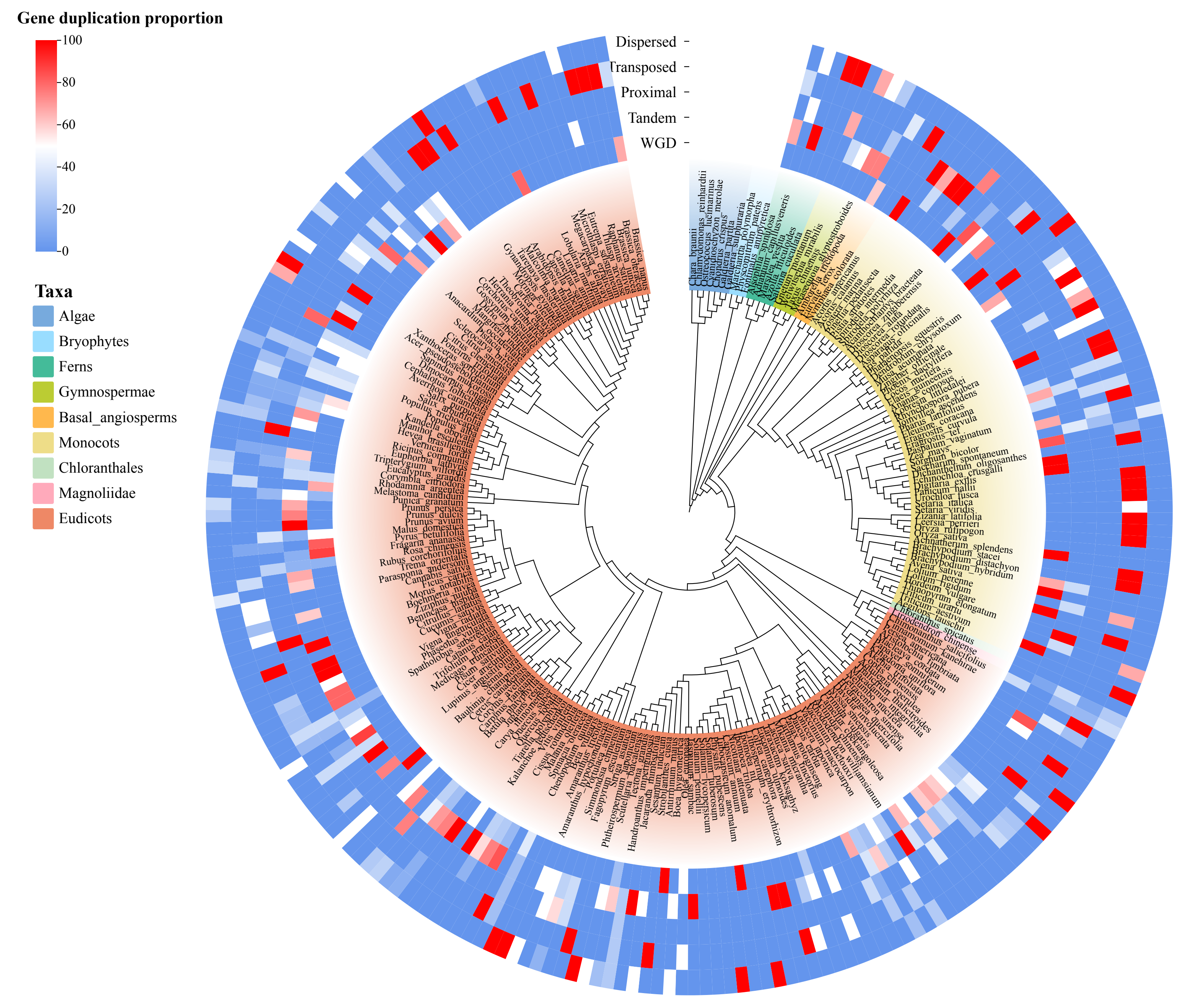

Gene duplication proportion
100
80
60
40
20
0
Taxa
Algae
Bryophytes
Ferns
Gymnospermae
Basal_angiosperms
Monocots
Chloranthales
Magnoliidae
Eudicots
Dispersed
Transposed
Proximal
Tandem
WGD
