# Supplementary figures and images for: A Comprehensive Evolutionary Analysis of the Dihydroflavonol 4-Reductase (DFR) Gene Family in Plants: Insights from 237 Species
Source: Genes (Basel). 2025 Mar 29;16(4):396. doi: 10.3390/genes16040396 (PMC12027299; doi:10.3390/genes16040396)

Tree scale 0.6

Type

Asn

Asp

GroupI

GroupII

GroupIII

GroupIV

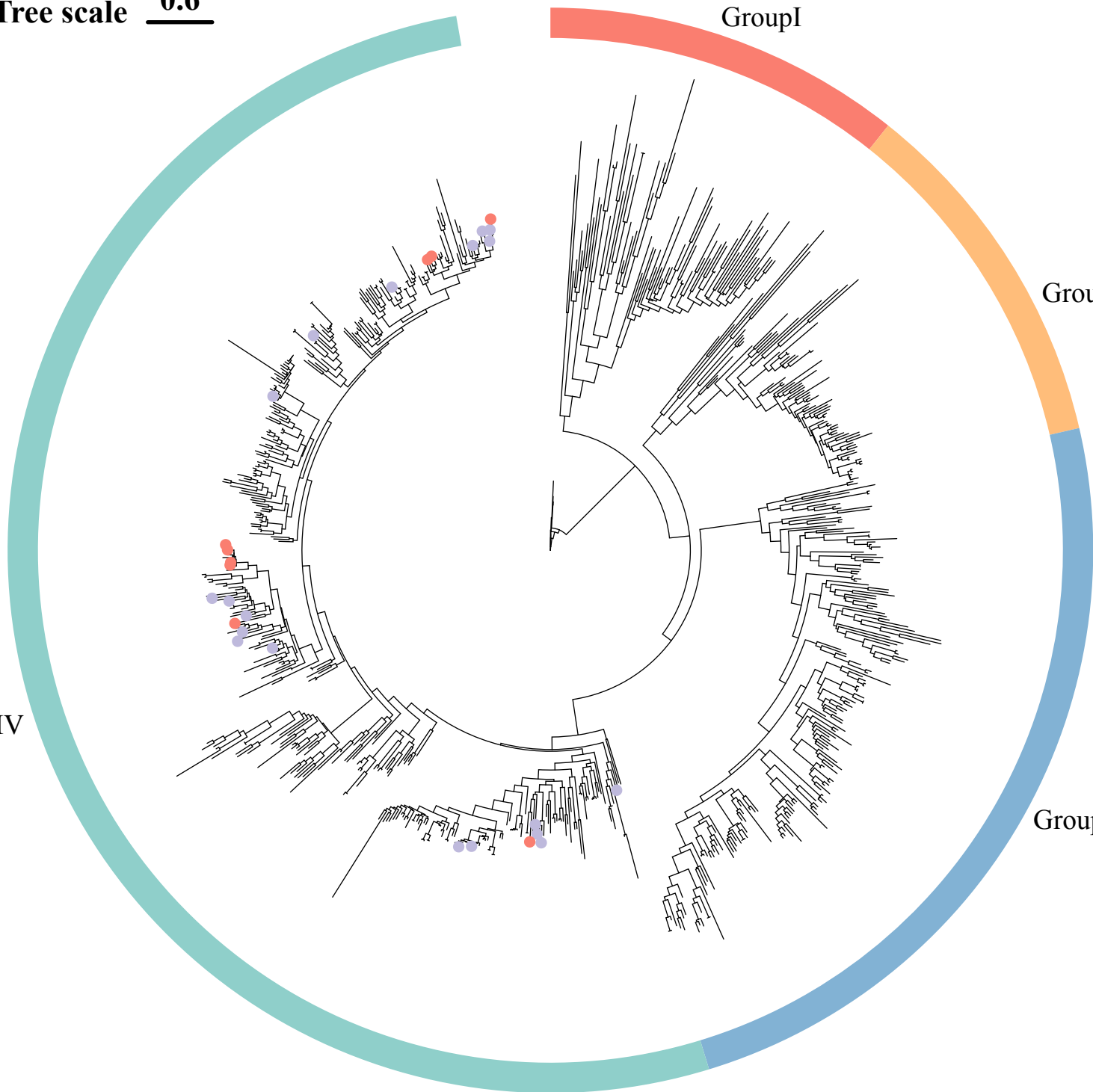

Supplement: Supplementary file 1 [file genes-16-00396-s001.zip › supplementary material/Figure S2.pdf]

motif\_1

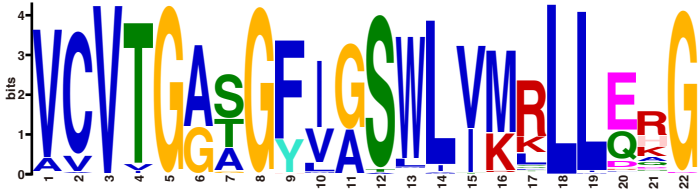

motif\_2

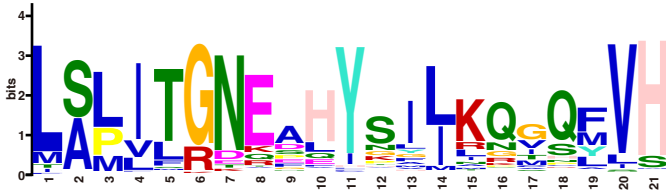

motif\_3

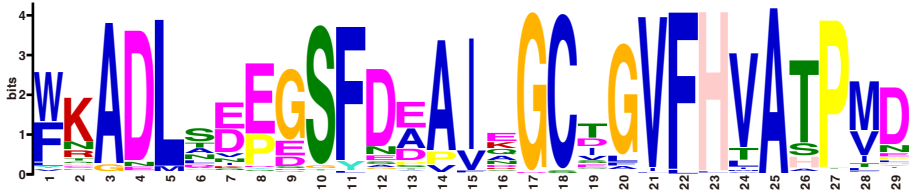

motif\_4

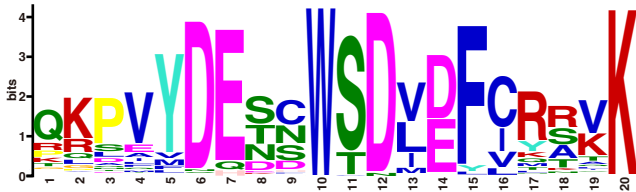

motif\_5

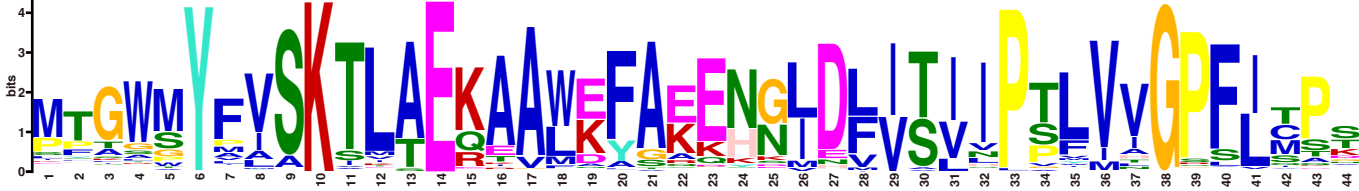

motif\_6

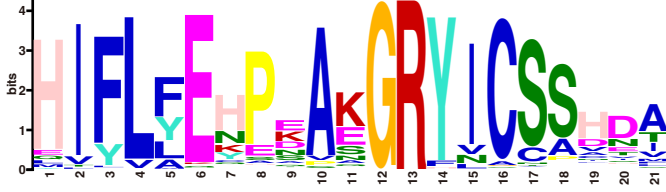

motif\_7

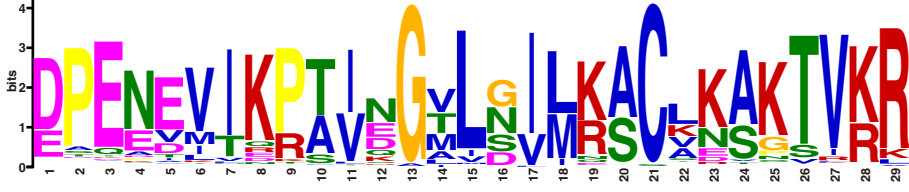

motif\_8

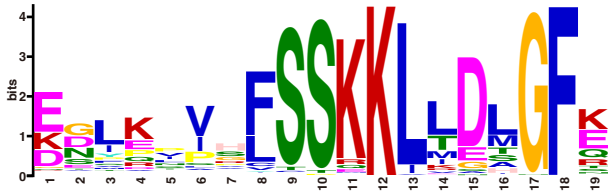

motif\_9

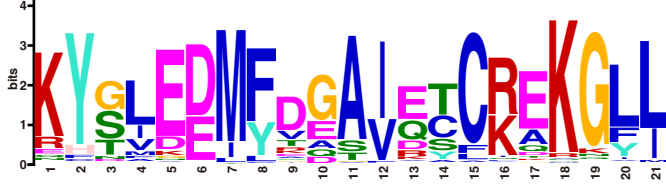

motif\_10

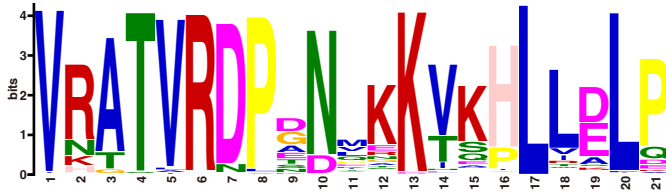

Supplement: Supplementary file 1 [file genes-16-00396-s001.zip › supplementary material/Figure S4.pdf]
